# Severe coagulopathy and inflammation occurred after resection of giant right ventricular intimal sarcoma with cardiopulmonary bypass: a case report

**DOI:** 10.1186/s12871-024-02416-w

**Published:** 2024-01-31

**Authors:** Menghan Liu, Xuejie Li, Ronghua Zhou

**Affiliations:** Department of Anesthesiology, West China Hospital, Sichuan University, Chengdu, 610041 China

**Keywords:** Primary cardiac intimal sarcoma, Cardiopulmonary bypass, Contact activation, Coagulopathy, Inflammation

## Abstract

**Background:**

Primary malignant cardiac tumors are rare in clinic, and surgical resection under cardiopulmonary bypass (CPB) remains the main treatment. The non-physiological perfusion process of CPB leads to contact activation, and the resulting coagulopathy and systemic inflammatory response syndrome (SIRS) are common complications. However, it is difficult to predict the impact of foreign tumor fragments on this pathophysiological process once they enter the bloodstream, making this phenomenon more complex and challenging.

**Case Presentation:**

We report a case of cardiac intimal sarcoma who developed severe coagulopathy and widespread inflammation after excision of massive right ventricular tumor and replacement of tricuspid valve by median sternotomy under CPB. Although the procedure was expected to cause tumor cell necrosis and precautions were taken, uncontrolled massive postoperative bleeding, persistent fever, abnormally elevated inflammatory markers, and recurrent malignant arrhythmias occurred after surgery. In addition to common factors, the most possible underlying mechanism is contact activation triggered following surgical procedure for intimal sarcoma with CPB.

**Conclusion:**

Patients with intracardiac malignant tumors are at a high risk for serious contact activation during CPB. Preventive application of comprehensive anti-inflammatory measures such as drugs and adsorptive CPB technology, as well as point-of-care (POC) monitoring of coagulation status will be helpful for individualized guidance and optimization of CPB management, and improvement of patient prognosis.

## Background

Primary intimal sarcomas are malignant mesenchymal tumors originating from vascular intima and usually involving great vessels. Cardiac intimal sarcomas are extremely rare and have a poor prognosis, characterized by spindle and pleomorphic cell clusters that express both MDM2 and CDK4 [1]. Radical surgical resection to obtain a negative margin remains the only definitive mode of treatment.

Cardiopulmonary bypass (CPB), a routine adjunct to open-heart surgery, can provoke abnormal coagulation and inflammation. Most patients will experience transient laboratory abnormalities after surgery, but in extreme cases there will be a series of major complications that seriously affect the prognosis. Previous studies have revealed potential triggers for CPB-associated inflammation and coagulopathy, including extensive surgical trauma, prolonged contact of blood components with the artificial surface of the CPB circuit, ischemia-reperfusion injury, hypothermia, hemodilution, platelet activation and dysfunction, inappropriate use of heparin and protamine, and so on [2–4]. But there is still no single effective intervention to prevent the activation of these two interacting pathways. While in patients with tumors, it has been suggested that the tumor lysis syndrome (TLS) may occur after open-heart surgery [5]. During TLS, a large number of tumor cells rapidly lyse to form tumor cell fragments and release metabolites into circulation, which may be involved in promoting contact activation and exacerbating systemic inflammatory system and coagulation system disorders.

Here, we report a unique case of resection of giant right ventricular intimal spindle cell sarcoma with CPB, which was complicated with severe coagulapathy and inflammation, and frequent malignant arrhythmias postoperatively. After comprehensive treatment, the patient was finally discharged 18 days after surgery.

## Case presentation

**Fig. 1:**
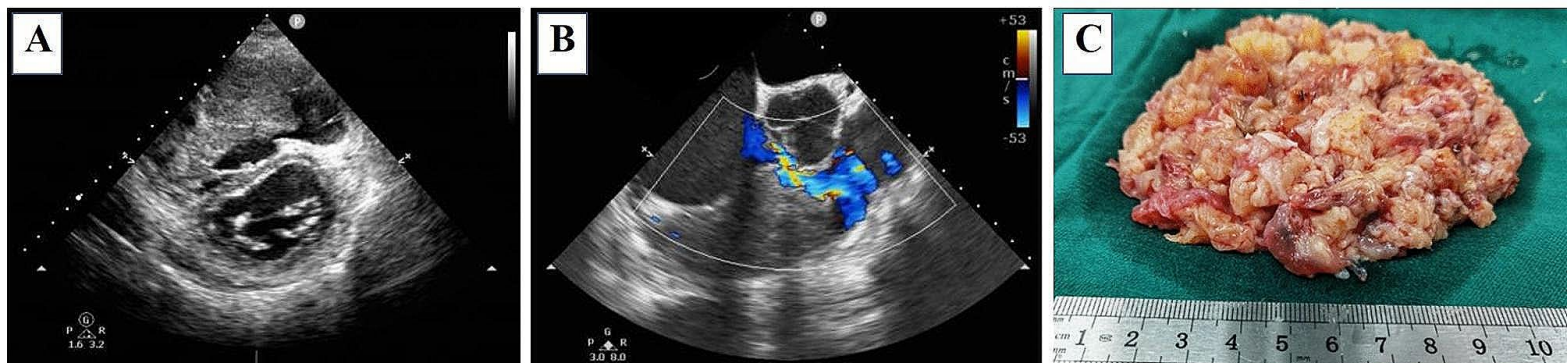
(**A**) and (**B**) represent transthoracic short axis section of LV and the mid-esophageal RV inflow-outflow view. Both TTE and TEE showed a huge mass occupying the inflow tract and most of the right ventricular cavity. The mass fused and wrapped with the tricuspid valve, and the demarcation between the tumor and the myocardium was not clear. The patient had a narrow gap to provide forward blood flow during diastole. (**C**) shows the excised fragmented tumor tissue

A 43-year-old previously healthy male patient, weighing 70 kg, was admitted to hospital due to facial edema in the past two months and aggravation of lower extremity edema recently accompanied by decreased activity endurance. Transthoracic echocardiography revealed a 90 × 65 × 52 mm large solid mass filled the right ventricular outflow tract and most of the right ventricular cavity, which fused and wrapped with the anterior and posterior leaflets of tricuspid valve, and only septal leaflet activity was seen (Fig. 1A). And a smaller one of about 20 × 17 × 25 mm occupied the apex. The left ventricular function is normal, with an ejection fraction of 60%. Preoperative routine laboratory examinations exhibited only mild thrombocytopenia with a platelet count of 83 × 10^9^ /L. There were no obvious abnormalities in liver and kidney function, coagulation function, and inflammatory markers.

On the day of surgery, transesophageal echocardiography (TEE) further confirmed the mass was approximately 90 mm in size and the tricuspid valve was seriously involved (Fig. 1B). CPB was performed using a roller pump, hollow-fiber oxygenator, and moderate hypothermia. Two filters were placed in the CPB circuit to prevent possible tumor debris and microemboli from entering the circulation. The circuit was primed with 200 mL of Lactate Ringer’s solution, 1000 mL of Succinylated Gelatin Injectiol and 3,750 U of heparin. 27,500 U (400 U/kg) of unfractionated heparin was injected intravenously after thoracotomy, with a resultant activated clotting time (ACT) value of 999 s observed. CPB was then commenced, with the flow rate of 2.4–2.6 L/(min·m^2^). After opening the right atrium, the tumor was noted to infiltrate into the endocardium and myocardium, and extend to the anterior, posterior, and septal leaflets of the tricuspid valve. An en-bloc resection was not possible, but an aggressive debulking procedure was performed. The resected tumor fragments are shown in Fig. 1C. Then, under small-dose vasoactive drugs and inotropic support with epinephrine (0.03 µg/kg/min) and norepinephrine (0.06 µg/kg/min), the patient was gradually weaned from CPB with normal heart rate (89 bpm), rhythm and blood pressure (101/56mmHg). TEE showed normal systolic function of both ventricles and normal left ventricular end-diastolic diameter. The total CPB time was 211 min with a cross-clamp time of 162 min. A total of 250 mg protamine was used to neutralize heparin (the ratio of protamine to heparin was 0.55:1), and the corresponding ACT value was 216 s, which was equivalent to the basic ACT value of 213 s.

However, the patient’s hemodynamics fluctuated greatly before leaving the operating room, and the drainage volume was up to 200 mL within 10 min. Hemodynamics could only be maintained under large-dose vasoactive drugs and inotropic support with milrinone (0.5 µg/kg/min), epinephrine (0.04 µg/kg/min), norepinephrine (0.75 µg/kg/min), m-hydroxylamine (0.75 µg/kg/min) and pituitrin (5U/h). The surgeons immediately performed a second thoracotomy for exploration, but no obvious surgical bleeding points were found except for extensive oozing in the surgical area. Severe coagulopathy and even acute disseminated intravascular coagulation (DIC) were highly suspected. The result of the thromboelastography (TEG) showed that R time and K time were prolonged, and alpha angle and maximum amplitude (MA) were decreased, suggesting coagulation factors, fibrinogen and platelet deficiency or insufficiency. The patient received intravenous infusion of 4 U of packed red cells, 950 mL of fresh frozen plasma, 1 g of fibrinogen, 600 U of prothrombin complex concentrate, 20 g of 20% albumin within two hours. Besides, 1g and 1.5 g of tranexamic acid were applied before and after rethoracotomy for hemostasis. Hemodynamics gradually stabilized with the support of smaller doses of norepinephrine (0.2 µg/kg/min) and pituitrin (3U/h) when the patient was transferred to intensive care unit (ICU).

**Fig. 2:**
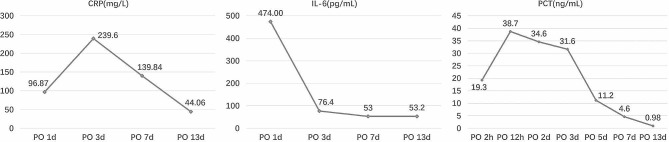
Laboratory indicators showed an abnormal increase in postoperative inflammatory markers in this patient. The inflammatory response was the most serious in the first three days after operation, and then gradually improved, but it did not fall to the normal value in the end. *CRP* C-reactive protein, *IL-6* interleukin-6, *PCT* procalcitonin

The results of the blood samples taken by the patient upon entering the ICU showed thrombocytopenia with a platelet count of 38 × 10^9^/L and moderate anemia with a hemoglobin level of 78 g/L, and TEG test showed no significant improvement compared with that measured intraoperatively. Therefore, the patient received 10 U of cryoprecipitate, 2 U of platelets, and 3.5 U of packed red cells. Besides, the patient’s inflammatory markers increased significantly after surgery and peaked within three days, and did not return to normal until the patient was discharged from the hospital, with peak interleukin-6 of 474.00 pg/mL and procalcitonin of 38.7 ng/mL on postoperative day1 (POD1), and peak C-reactive protein of 239.6 mg/L on POD3. The evolution of postoperative inflammatory markers is shown in Fig. 2. From POD2 to POD6, the patient experienced recurrent moderate-to-high fevers up to 39.6℃ despite attempted at active cooling. As the blood culture was negative, antibiotic therapy was discontinued. At the same time, the patient suffered from frequent atrial fibrillation, ventricular tachycardia, and supraventricular tachycardia from POD3 to POD6, and was treated with antiarrhythmic drugs and electrocardioversion. The patient was transferred back to the general ward on POD9 and discharged on POD18 after his condition stabilized. Postoperative supplementary pathological reports supported intimal sarcoma.

## Discussion and conclusions

Primary cardiac intimal sarcomas tend to occur in left atrium, [1] but have been reported in all four cardiac chambers. Intimal sarcoma is a kind of undifferentiated invasive sarcoma characterized by overexpression and amplification of MDM2, [1] but early diagnosis is difficult due to its low incidence, insidious and non-specific symptoms. The prognosis of these patients remains dismal. Although adjuvant chemotherapy may improve the survival of patients, radical surgical resection under CPB to obtain a negative incisal margin is still the only definitive treatment [1, 6, 7]. 

Our patient initially presented with progressive lower limb edema and fatigue, but did not exhibit specific cardiac or embolism-related symptoms. In order to improve the patient’s outcomes, a right ventricular massive mass resection was performed after adequate preoperative preparation. The procedure was uneventful, but the patient experienced uncontrolled massive bleeding, persistent fever and abnormal elevated inflammatory markers, and recurrent malignant arrhythmias after surgery. Although spontaneous DIC may have occurred in our patient, it seems more reasonable to speculate that the massive destruction of tumor cells during surgery, combined with the prolonged use of CPB, may have triggered cytokine storms leading to severe coagulopathy, SIRS and electrical storms.

Coagulopathy after CPB is very common in most cardiac surgeries and is generally multifactorial. Extensive surgical trauma, prolonged contact of blood with the artificial surface of the CPB circuit, unfractionated heparin residue, inappropriate use of protamine, hypothermia, platelet activation and dysfunction, hyperfibrinolysis and inflammatory cascade are all possible triggers.

The inflammatory response induced by CPB is also inevitable. The early phase is believed to be caused by the contact of blood with the artificial material of the CPB circuit. Ischemia/reperfusion injury and endotoxemia further trigger the late phase through leucocyte dependent and non-leucocyte dependent responses. Surgical trauma is also a potential cause. SIRS will eventually occur when the body’s homeostasis is destroyed [8]. 

Notably, the contact activation caused by blood exposure to the non-endothelial surface of CPB and the cascade of reactions it triggers play a key role in the systemic activation of the inflammatory and hemostatic systems (fibrin formation, platelet activation/consumption, and endothelial damage) during CPB. According to the Vroman effect, when blood comes into contact with the non-physiological surface of the CPB circuit, plasma proteins are sequentially absorbed and form a monolayer, which in turn mediates the activation of a series of humoral and cellular components through changes in protein conformation [9]. Activation of the contact system is accompanied by activation of factor XII-X and further leads to the production of bradykinin and kallikrein, which directly activates the intrinsic coagulation pathway and neutrophils. In addition, the complement system, fibrinolytic system, platelets, endothelial cells, monocytes, and lymphocytes are also indirectly activated. The production of Xa further induces the production of thrombin and leads to widespread activation of both intrinsic and extrinsic coagulation systems [8–10]. The activation of the above five plasma protein systems and blood cells causes extensive cross-talk between the inflammation and coagulation systems, resulting in the continuous consumption of clotting factors and platelets, bleeding, thrombosis, and the release of various cytokines during CPB, which may ultimately leads to end organ dysfunction and other potential postoperative complications.

The entry of tumor debris into the bloodstream is likely to exacerbate the contact activation process. On the one hand, as for patients with tumors, coagulopathy are more likely to occur. One possible hypothesis is the Kasabach Merritt phenomenon (KMP), which is characterized by severe thrombocytopenia [11, 12]. In addition, experimental studies also show that solid tumors and damaged endothelial cells may express procoagulant molecules (tissue factors and cancer procoagulants), or express high levels of fibrinolytic inhibitor PAI-1 to induce a hypofibrinolytic state, or, alternatively, express plasminogen-activating factors (urokinase-type plasminogen activator and tissue-type plasminogen activator), which may lead to hyperfibrinolytic state [13, 14]. Some cases of DIC associated with angiosarcoma have been reported [11–13, 15–17]. On the other hand, a large number of pro-inflammatory cytokines such as interleukin-6 (IL-6), IL-8, IL-1 and tumor necrosis factor α (TNF-α) are released during rapid cell lysis, further exacerbating systemic inflammatory response [5]. The postoperative inflammation markers of this patient, especially procalcitonin, were significantly higher than those of non-tumor patients in our institution during the same period (38.7 ng/mL vs. 0.19 ng/mL) and those reported in previous literature [18]. 

Tumor lysis syndrome (TLS) is characterized by severe electrolyte disturbance, metabolic acidosis, acute renal impairment, multiple organ failure, and even death [5]. Our patient’s maximum serum potassium was 5.85 mmol/L, the minimum serum calcium was 0.928 mmol/L, and there were no obvious abnormalities in phosphorus and uric acid. Although our patient did not meet the diagnostic criteria for TLS and did not present with the above clinical extremes, the presence of tumolytic processes is highly suspected in combination with the abnormal coagulation, inflammation, and electrical storm in our patient.

This case highlights that the degree of contact activation during cardiac surgery with CPB in patients with malignant tumors may be much greater than expected, further complicating CPB-associated inflammation and coagulopathy. Therefore, it is necessary to pay great attention to such patients and take preventive measures. First of all, identifying high-risk patients is a priority, including malignant tumors, severe infections, severe liver dysfunction, and so on. Secondly, it should be equipped with primary filtration devices. It seems futile to install two blood reservoirs in advance from this case, so more advanced adsorptive blood purification devices may be necessary, such as CytoSorb. Based on its non-selective adsorption properties, concentration dependence, large adsorption area, and easy assembly in the CPB circuit, CytoSorb can efficiently adsorb cytokines between 5 and 60 kDa in size and has shown optimistic results in cardiac surgery, especially in high-risk groups [19]. Thirdly, in cases where CPB cannot be avoided, a combination of pharmacological and technical anti-inflammatory strategies may be useful. Corticosteroids (dexamethasone), serine protease inhibitors (aprotinin), antioxidants (mannitol, allopurinol, and N-acetyl cysteine), complement inhibitors (pexilizumab), phosphodiesterase inhibitors (milrinone), and other pharmacologic drugs have been investigated as potential anti-inflammatory agents. Technical strategies such as heparin-bonded circuits, ultrafiltration and leukocyte filtration may beneficial [20]. Fourthly, real-time and accurate monitoring of coagulation is very important. There is evidence to support the benefits of rotational thromboelastometry (ROTEM) and TEG as point-of-care (POC) tools to assess perioperative coagulation [21, 22]. The use of POC hemostasis test can comprehensively detect the coagulation function, analyze the causes of bleeding, timely and targeted intervention, and guide component blood transfusion.

In conclusion, patients with intracardiac tumors need careful preoperative evaluation and adequate CPB preparations to minimize contact activation. Effective monitoring methods and preventive measures are very important. The advantages of the rational application of anti-inflammatory drugs and adsorptive devices to reduce the inflammatory response and POC monitoring of coagulation status to guide the reconstruction of coagulation function in CPB cannot be ignored.

## Data Availability

No datasets were generated or analysed during the current study.

## Abbreviations

CPB: Cardiopulmonary bypass
DIC: Disseminated intravascular coagulation
KMP: Kasabach Merritt phenomenon
MA: Maximum amplitude
POC: Point-of-care
POD: Postoperative day
ROTEM: Rotational thromboelastometry
SIRS: Systemic inflammatory response syndrome
TEG: Thromboelastography
TLS: Tumor lysis syndrome

## References

[1] Neuville A, Collin F, Bruneval P, Parrens M, Thivolet F, Gomez-Brouchet A, Terrier P, de Montpreville VT, Le Gall F, Hostein I, Lagarde P, Chibon F, Coindre JM (2014). Intimal sarcoma is the most frequent primary cardiac sarcoma: clinicopathologic and molecular retrospective analysis of 100 primary cardiac sarcomas. Am J Surg Pathol.

[2] Paparella D, Yau TM, Young E (2002). Cardiopulmonary bypass induced inflammation: pathophysiology and treatment. An update. Eur J Cardiothorac Surg.

[3] Paparella D, Brister SJ, Buchanan MR (2004). Coagulation disorders of cardiopulmonary bypass: a review. Intensive Care Med.

[4] Day JRS, Taylor KM (2005). The systemic inflammatory response syndrome and cardiopulmonary bypass. Int J Surg.

[5] Shih JM (2021). Tumor lysis syndrome following Thoracotomy under Cardiopulmonary Bypass in a case of Hepatocellular Carcinoma with Right Atrial and Inferior Vena Cava Tumor Thrombus. Cureus.

[6] Andersen KF, Someh NS, Loft A, Brittain JM (2020). Primary Cardiac Intimal Sarcoma visualized on 2-[18F]FDG PET/CT. Diagnostics.

[7] Tyebally S, Chen D, Bhattacharyya S, Mughrabi A, Hussain Z, Manisty C, Westwood M, Ghosh AK, Guha A (2020). Cardiac tumors. JACC: CardioOncology.

[8] Warren OJ, Smith AJ, Alexiou C, Rogers PLB, Jawad N, Vincent C, Darzi AW, Athanasiou T (2009). The inflammatory response to cardiopulmonary bypass: part 1—Mechanisms of Pathogenesis. J Cardiothorac Vasc Anesth.

[9] Hatami S, Hefler J, Freed DH (2022). Inflammation and oxidative stress in the context of extracorporeal Cardiac and Pulmonary Support. Front Immunol.

[10] Ranucci M, Baryshnikova E (2019). Inflammation and coagulation following minimally invasive extracorporeal circulation technologies. J Thorac Dis.

[11] Honda K, Ando M, Sugiyama K, Mitani S, Masuishi T, Narita Y, Taniguchi H, Kadowaki S, Ura T, Muro K (2017). Successful Treatment of Cardiac Angiosarcoma Associated with disseminated intravascular coagulation with Nab-Paclitaxel: a Case Report and Review of the literature. Case Rep Oncol.

[12] Farid M, Ahn L, Brohl A, Cioffi A, Maki RG. Consumptive coagulopathy in angiosarcoma: a recurrent phenomenon? Sarcoma 2014; 2014: 617102. 10.1155/2014/617102 PMC3945465 24693222

[13] Bien E, Maciejka-Kapuscinska L, Niedzwiecki M, Stefanowicz J, Szolkiewicz A, Krawczyk M, Maldyk J, Izycka-Swieszewska E, Tokarska B, Balcerska A (2010). Childhood rhabdomyosarcoma metastatic to bone marrow presenting with disseminated intravascular coagulation and acute tumour lysis syndrome: review of the literature apropos of two cases. Clin Exp Metastasis.

[14] Levi M (2019). Disseminated intravascular coagulation in Cancer: an update. Semin Thromb Hemost.

[15] Yagi T, Nakamura H, Wakamatsu T, Imura Y, Tamiya H, Sabe H, Yamashita K, Watanabe M, Takenaka S (2021). Primary breast angiosarcoma with disseminated intravascular coagulation is successfully treated with self-subcutaneous unfractionated heparin calcium injection: a case report. Mol Clin Oncol.

[16] Fernando N, Butcherine K, Harle R, Ritchey D (2019). Mysterious abdominal pain and disseminated intravascular coagulation due to hepatic angiosarcoma. Intern Med J.

[17] Paul R, Morgan D, Levitt M, Baker R (2012). Acute disseminated intravascular coagulation following surgical resection of a myeloid sarcoma in a 57-year-old male. Clin Pract.

[18] Bruins P, Te VH, Yazdanbakhsh AP, Jansen PG, van Hardevelt FW, de Beaumont EM, Wildevuur CR, Eijsman L, Trouwborst A, Hack CE (1997). Activation of the complement system during and after cardiopulmonary bypass surgery: postsurgery activation involves C-reactive protein and is associated with postoperative arrhythmia. Circulation.

[19] Liu MH, Yu H, Zhou RH. Application of Adsorptive Blood Purification Techniques during Cardiopulmonary Bypass in Cardiac Surgery. Oxid Med Cell Longev 2022; 2022: 6584631. 10.1155/2022/6584631 PMC9159835 35663201

[20] Warren OJ, Watret AL, de Wit KL, Alexiou C, Vincent C, Darzi AW, Athanasiou T (2009). The inflammatory response to cardiopulmonary bypass: part 2—Anti-Inflammatory therapeutic strategies. J Cardiothorac Vasc Anesth.

[21] Wikkelso A, Wetterslev J, Moller AM, Afshari A. Thromboelastography (TEG) or thromboelastometry (ROTEM) to monitor haemostatic treatment versus usual care in adults or children with bleeding. Cochrane Database Syst Rev 2016: CD007871. 10.1002/14651858.CD007871.pub3 PMC6472507 27552162

[22] Da LL, Nascimento B, Shankarakutty AK, Rizoli S, Adhikari NK (2014). Effect of thromboelastography (TEG(R)) and rotational thromboelastometry (ROTEM(R)) on diagnosis of coagulopathy, transfusion guidance and mortality in trauma: descriptive systematic review. Crit Care.

